# The Dr. Nathan Smith Davis Memorial

**Published:** 1908-04

**Authors:** J. T. Wilson

**Affiliations:** Sherman, Texas


### The Dr. Nathan Smith Davis Memorial.

*Editor Texas Medical Journal.*

DEAR SIR: Please publish in your April issue the following acknowledgment of the contributions to the Dr. Nathan Smith Davis Memorial Fund by the County Societies named in the list:

Bastrop, $3.25; Baylor, $2; Bell, $5; Brown, $1.25; Collin, $4.75; Comal, $1; Denton; $2; El Paso, $13.25; Fayette, $1; Grayson, $16.25; Hill, $5; Hood, $1; Hunt, $5; Jasper-Newton, $1.50; Jefferson, $5; Kaufman, $9.25; Lee, $1.50; Red River, $2; Smith, $1.50; Tarrant, $23.50; Titus, $1.75; Travis, $11. The total amount received to date being $117.75.

I desire in this connection to thank the above list of county medical societies for their liberal contributions. It was hoped that Texas would contribute at least $250 to this fund; only $132.25 is needed to complete the amount. There is an estimate of 5000 regular physicians in the State and a contribution of 10 cents per capita by one-third of this number would be more than the desired amount. The members of the auxiliary committee in Texas are the Councilors of the Medical Association of the State. A report will be made to the American Medical Association at its meeting in June. It is probable that the character of the memorial will be determined upon then, as it is expected that the fund will be sufficient.

J. T. WILSON.

Sherman, Texas.

